# Lenalidomide–rituximab with high-dose methotrexate for treatment of patients with newly diagnosed primary cns lymphoma: a promising first-line approach

**DOI:** 10.1007/s00277-025-06593-7

**Published:** 2025-10-02

**Authors:** Xiaoli Chang, Huanyuan Wang, Yixian Guo, Qiang Ma, Zhilian Zhao, Dongmei Zou, Jing Ni, Ronghua Hu, Hong Zhao, Wuhan Hui, Li Su, Wanling Sun

**Affiliations:** 1 Department of Hematology, Xuanwu Hospital, National Center for Neurological Disorders, National Clinical Research Center for Geriatric Diseases, Capital Medical University, No.45 Changchun Street, Xicheng District, Beijing, China; 2 Comprehensive Center for Neuro-oncology, Xuanwu Hospital, Capital Medical University, Beijing, China; 3 Beijing Municipal Geriatric Medical Research Center, Beijing, China; 4 Clinical Laboratory, Department of Hematology, Xuanwu Hospital, Capital Medical University, Beijing, China; 5 Department of Radiology and Nuclear Medicine, Xuanwu Hospital, Capital Medical University, Beijing, China

**Keywords:** Primary central nervous system lymphoma, Lenalidomide, First-line therapy, Efficacy, Adverse effects

## Abstract

Primary central nervous system lymphoma (PCNSL) is a rare and aggressive malignancy with limited treatment options and presents significant therapeutic challenges. Although high-dose methotrexate (HD-MTX)-based immunochemotherapy, followed by autologous stem cell transplantation (ASCT), improves outcomes in patients with PCNSL, in clinical practice, ASCT eligibility is frequently restricted by medical unsuitability, patient choice, and socioeconomic factors. In this retrospective study, we evaluated 12 newly diagnosed PCNSL patients who were treated with a combination of rituximab, HD-MTX, and lenalidomide (R2-MTX) without ASCT. With a median follow-up of 43.0 months, the R2-MTX regimen demonstrated superior clinical efficacy, achieving an overall response rate (ORR) of 91.7% (95% CI: 61.5–99.8%), with a complete response (CR) rate of 66.7% at the end of induction therapy. The median overall survival (OS) was not reached, while the median progression-free survival (PFS) was 62 months (range: 6–64 months). The estimated 2- and 5-year OS rates were 91.7% (95% CI: 76.0–100%) and 70.7% (95% CI: 52.1–99.3%), respectively, with corresponding PFS rates of 66.7% (95% CI: 50.1–93.3%) and 57.1% (95% CI: 34.5–83.7%), respectively. Treatment-related toxicities were manageable, with no grade ≥ 3 adverse events observed. The most common adverse effect was neutropenia (46.2%). Notably, patients with CARD11 mutations experienced a high rate of early relapse despite lenalidomide treatment. In conclusion, the R2-MTX regimen showed encouraging efficacy and a manageable safety profile in a small cohort of newly diagnosed PCNSL patients unsuitable for ASCT. These preliminary findings suggest that R2-MTX may be a promising therapeutic alternative, but validation in larger, prospective multicenter studies is warranted.

## Introduction

Primary central nervous system lymphoma (PCNSL) is a rare aggressive non-Hodgkin’s lymphoma (NHL) that is associated with a relatively poor prognosis, with a 5-year survival rate of only 30–40% [1, 2]. Approximately 10–30% of patients are resistant to high-dose methotrexate (HD-MTX)-based induction therapy, and nearly 50–60% of patients experience recurrence after the initial response [3, 4]. Although autologous haematopoietic stem cell transplantation (ASCT) can improve the prognosis of PCNSL patients, patients who are ineligible for ASCT still have extremely poor outcomes [5]. Furthermore, the applicability of ASCT in clinical practice is often restricted by medical comorbidities, patient preference, and socioeconomic barriers. Lenalidomide is a potent antiproliferative and immunomodulatory agent that can penetrate ventricular cerebrospinal fluid (CSF) [6]. It has pleiotropic antitumour effects and cell-autonomous cytotoxic effects on lymphoid tumours and has shown promise in patients with relapsed/refractory (R/R) PCNSL [7, 8]. In addition, lenalidomide enhances the antibody-dependent cell-mediated cytotoxicity of rituximab and may overcome rituximab resistance in patients with NHL [9, 10]. Preclinical data indicate a synergistic effect between lenalidomide and rituximab (R2) [11]. Phase I and II studies of rituximab and lenalidomide in combination demonstrated similar favourable results in patients with relapsed/refractory PCNSL [7, 8]. While the potential benefits of lenalidomide in newly diagnosed lymphoma patients remain underexplored, this study aimed to systematically evaluate the efficacy and safety of a novel combination regimen incorporating lenalidomide, rituximab, and HD-MTX in treatment-naive PCNSL patients.

## Materials and methods

### Patients

In this real-world study, we retrospectively analysed 12 consecutive immunocompetent adult patients with newly diagnosed PCNSL who were treated at the Department of Hematology, Xuanwu Hospital, Capital Medical University, between May 2019 and May 2022. All patients received R2-MTX induction therapy, having either been deemed ineligible for upfront ASCT by investigators or having declined the procedure. Patients who achieved complete response (CR) or partial response (PR) subsequently received lenalidomide maintenance therapy for 24 months.

The inclusion criteria included: (i) newly diagnosed, histologically confirmed PCNSL; (ii) age ≥ 18 years; (iii) ECOG performance status of 0–3; and (iv) ineligibility for ASCT. The exclusion criteria were as follows: (i) comorbid mental disorders, (ii) unstable systemic diseases, (iii) a history of thrombotic events, (iv) immunodeficient states, or (v) pregnancy. All patients met the World Health Organization (WHO) classification criteria for haematolymphoid tumours, with the diagnosis confirmed through an integrated pathological and clinical assessment. DLBCL was histologically verified via stereotactic biopsy of CNS lesions, which was supported by immunohistochemical profiling. Systemic staging excluded extraneural involvement through comprehensive evaluation, including (i) whole-body fluorodeoxyglucose positron emission tomography (FDG-PET) or contrast-enhanced CT, (ii) complete ophthalmic examination with slit-lamp evaluation, and (iii) bilateral bone marrow aspiration and biopsy. This retrospective analysis received approval from the Medical Ethics Committee of the Xuanwu Hospital, Capital Medical University. All patients provided written informed consent before treatment initiation and all procedures complied with the ethical standards outlined in the Declaration of Helsinki.

### Treatment regimen

Induction therapy comprised rituximab (375 mg/m² administered as an intravenous infusion on Day 0), HD-MTX (3.5 g/m² administered as a 3-hour intravenous infusion on Day 1), and lenalidomide (25 mg orally once daily on Days 1–21), with a total of 6 cycles repeated every 28 days. The methotrexate dose was reduced by one-third in patients meeting any of the following criteria: (1) age ≥ 70 years, (2) TT genotype of methylenetetrahydrofolate reductase (MTHFR), or (3) a history of transient liver and renal insufficiency. Maintenance therapy with lenalidomide monotherapy (25 mg/day for 21 days per 28-day cycle) was initiated for 24 months when patients achieved CR or PR after induction therapy. Intrathecal chemotherapy was not administered during induction phase. During the maintenance phase, intrathecal methotrexate (10 mg) was administered every 3 months.

### Efficacy and adverse effects

Tumour responses were comprehensively evaluated every two cycles using contrast-enhanced brain magnetic resonance imaging (MRI), cerebrospinal fluid (CSF) analysis, slit-lamp ocular examination, and whole-body CT. An interim assessment with FDG-PET was performed after four cycles. The therapeutic response after CR was evaluated every 3–6 months for 5 years. The efficacy endpoints included the ORR, PFS, and OS. The ORR included CR and PR according to the International Primary CNS Lymphoma Collaborative Group (IPCG) criteria. End of treatment (EOT) was defined as the completion of 24 months of the lenalidomide maintenance phase. AEs were systematically documented after each therapy cycle and graded according to the Common Terminology Criteria for Adverse Events (CTCAE) version 5.0.

### Genomic analysis

To characterize the genomic landscapes of the tumours, we performed next-generation sequencing (NGS) on available baseline tumour biopsy samples that were collected from the enrolled patients. The sequencing was performed using a targeted panel with an average coverage depth of approximately 1500×.

### Statistical analysis

Statistical analyses were performed using GraphPad Prism 8.0 and SPSS 22.0. Continuous variables (median [range] or mean ± SD) were compared using t tests or Mann‒Whitney U tests, as appropriate. Categorical variables (n [%]) were analysed by the χ² test or Fisher’s exact test. The 95% confidence intervals (CIs) for the binomial proportions were calculated using the exact Clopper‒Pearson method. Survival outcomes (PFS and OS) were assessed via Kaplan‒Meier analysis with 95% CIs.

## Results

### Patients

**Fig. 1:**
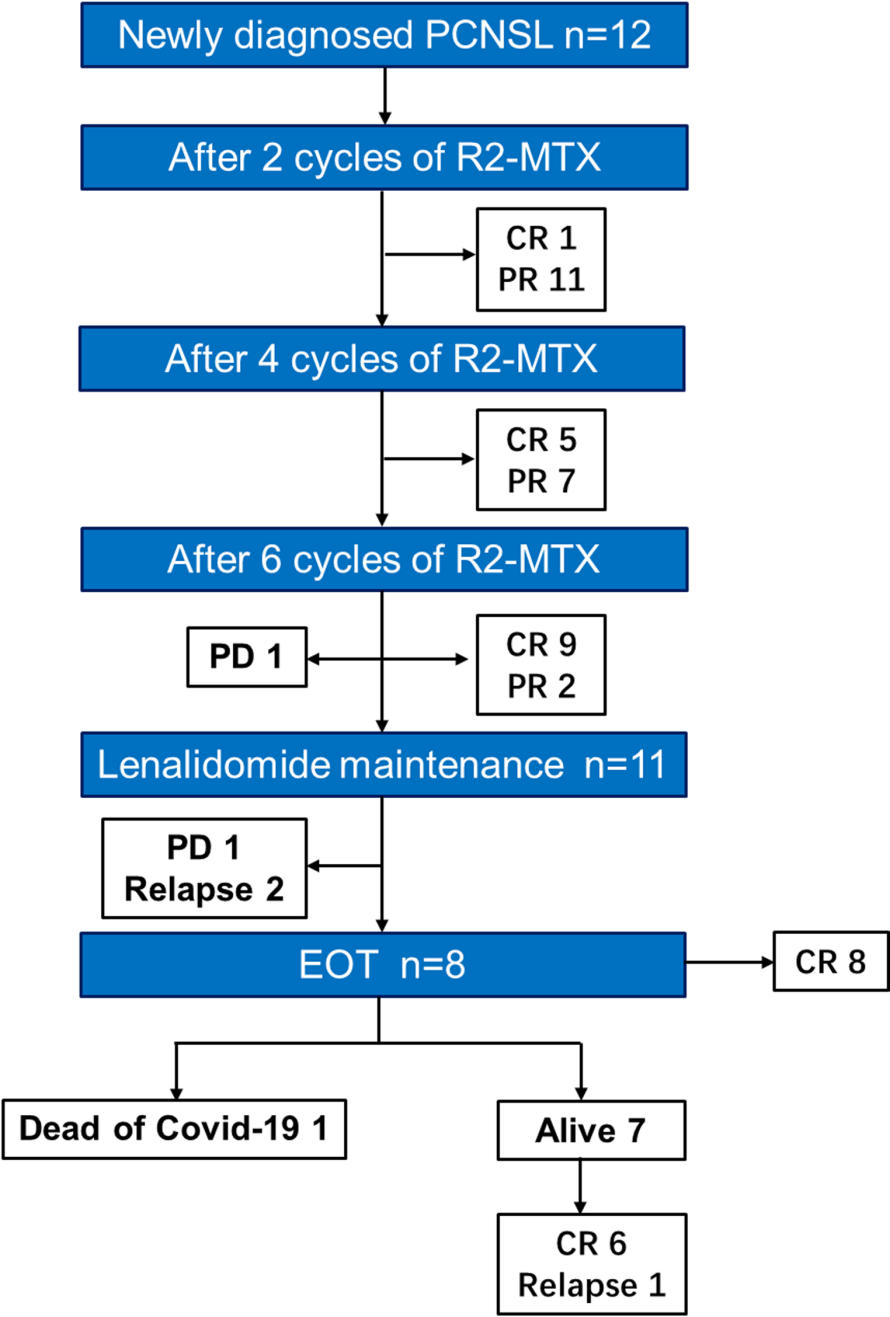
Flowchart outlining treatment outcomes. PCNSL, primary central nervous system lymphoma; R2-MTX, rituximab, methotrexate, and lenalidomide; CR, complete response; PR, partial response; PD, progressive disease

All 12 patients had DLBCL confined to the brain parenchyma, with neither CSF nor intraocular involvement detected at diagnosis. The flowchart of treatment outcomes is presented in Fig. 1. The majority of patients (11/12, 91.7%) completed R2-MTX induction therapy followed by lenalidomide maintenance therapy. The remaining patient experienced disease progression after six induction cycles and did not undergo maintenance therapy. The baseline demographic and clinical characteristics are detailed in Table 1.

**Table 1:** Baseline characteristics

Characteristics		R2-MTX (*n* = 12)
Age, years		
	Median (range)	53.0 (43–73)
Sex, n (%)		
	Males	7/12 (58.3)
	Females	5/12 (41.7)
ECOG, n (%)		
	0–1	1/12 (8.3)
	≥ 2	11/12 (91.7)
Histological subtypes, n (%)		
	GCB	2/12 (16.7)
	ABC	10/12 (83.3)
Prognostic score, n (%)		
IELSG		
	Low–intermediate risk	5 (41.7)
	High risk	7 (58.3)
MSKCC		
	Low–intermediate risk	1/12 (8.3)
	High risk	11/12 (91.7)
Induction therapy, n (%)		
	R2-MTX	12/12 (100)
Maintenance therapy, n (%)		
	Lenalidomide	11/12 (91.7)
	No maintenance	1/12 (8.3)

*ECOG *Eastern Cooperative Oncology Group; *GCB *germinal centre B cell; *IELSG *International Extranodal Lymphoma Study Group; *MSKCC *Memorial Sloan Kettering Cancer Center; *R2-MTX *rituximab, methotrexate, and lenalidomide

### Treatment efficacy

At the end of induction therapy, the ORR was 91.7% (95% CI: 61.5–99.8%). The median time to CR was 4 months (range: 2–9 months). Among the 12 patients, 11 (91.7%) proceeded to lenalidomide maintenance therapy. Notably, one of the two patients who achieved a PR after induction therapy subsequently achieved CR following 3 months of lenalidomide maintenance therapy. The detailed clinical responses are presented in Table 2.

**Table 2:** Clinical responses to treatments

	R2-MTX (*n* = 12)
Responses at the end of induction therapy, n (%)	
ORR	11/12 (91.7)
CR	9/12 (75.0)
PR	2/12 (16.7)
PD	1/12 (8.3)
Responses at the end of maintenance therapy, n (%)	
CR	8/12 (66.6)
PD/Relapse	4/12 (33.3)
Died of relapsed disease	2/12 (16.7)
Died of Covid-19	1/12 (8.3)
RDD of R2-MTX, n (%)	86.7
RDI of R2-MTX, n (%)	84.3
EOT, n (%)	8/12 (58.3)
Median duration of lenalidomide treatment (months)	30 (6–39)
Median PFS (months)	62 (6–64)
Median OS (months)	Not reached
2-y OS	91.7%
2-y PFS	66.7%
5-y OS	70.7%
5-y PFS	57.1%

*CR *complete response; *PR *partial response; *PD *progressive disease; *RDD *relative dose density; *RDI *relative dose intensity; *EOT *end of induction and maintenance treatment; *OS *overall survival; *PFS *progression-free survival

**Fig. 2:**
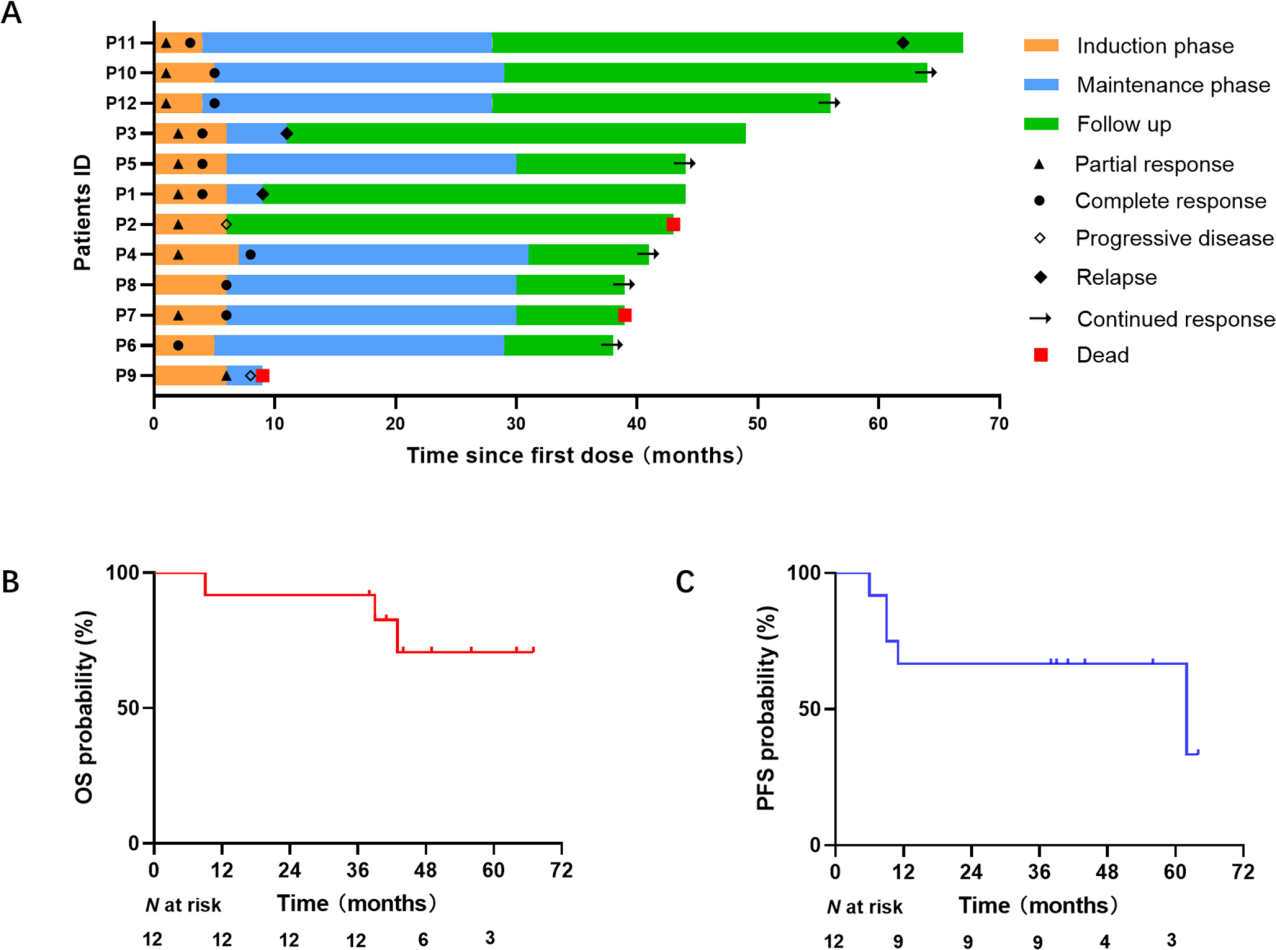
Clinical responses of newly diagnosed PCNSL patients. **A** Swimmer plot of R2-MTX treatment. Each bar line represents one patient; the length of the bar represents the time between the initiation of treatment and the last follow-up. **B** Kaplan‒Meier analysis of overall survival. **C** Kaplan‒Meier analysis of progression-free survival

Ultimately, disease progression (PD) or relapse occurred in 5 patients (41.7%), with a median time to PD/relapse of 9 months. Notably, one patient who achieved EOT experienced a late relapse 62 months after initial diagnosis. Three patients (25.0%) died—two from relapsed disease and one from COVID-19 after EOT (Fig. 2A).

With a median follow-up of 43.0 months (range: 2–67 months), the median overall survival (OS) was not reached. The median progression-free survival (PFS) was 62 months. The estimated 2- and 5-year OS rates were 91.7% (95% CI: 76.0–100%) and 70.7% (95% CI: 52.1–99.3%), respectively. The corresponding PFS rates were 66.7% (95% CI: 50.1–93.3%) at 2 years and 57.1% (95% CI: 34.5–83.7%) at 5 years (Fig. 2B and C).

### Genomic characteristics

**Fig. 3:**
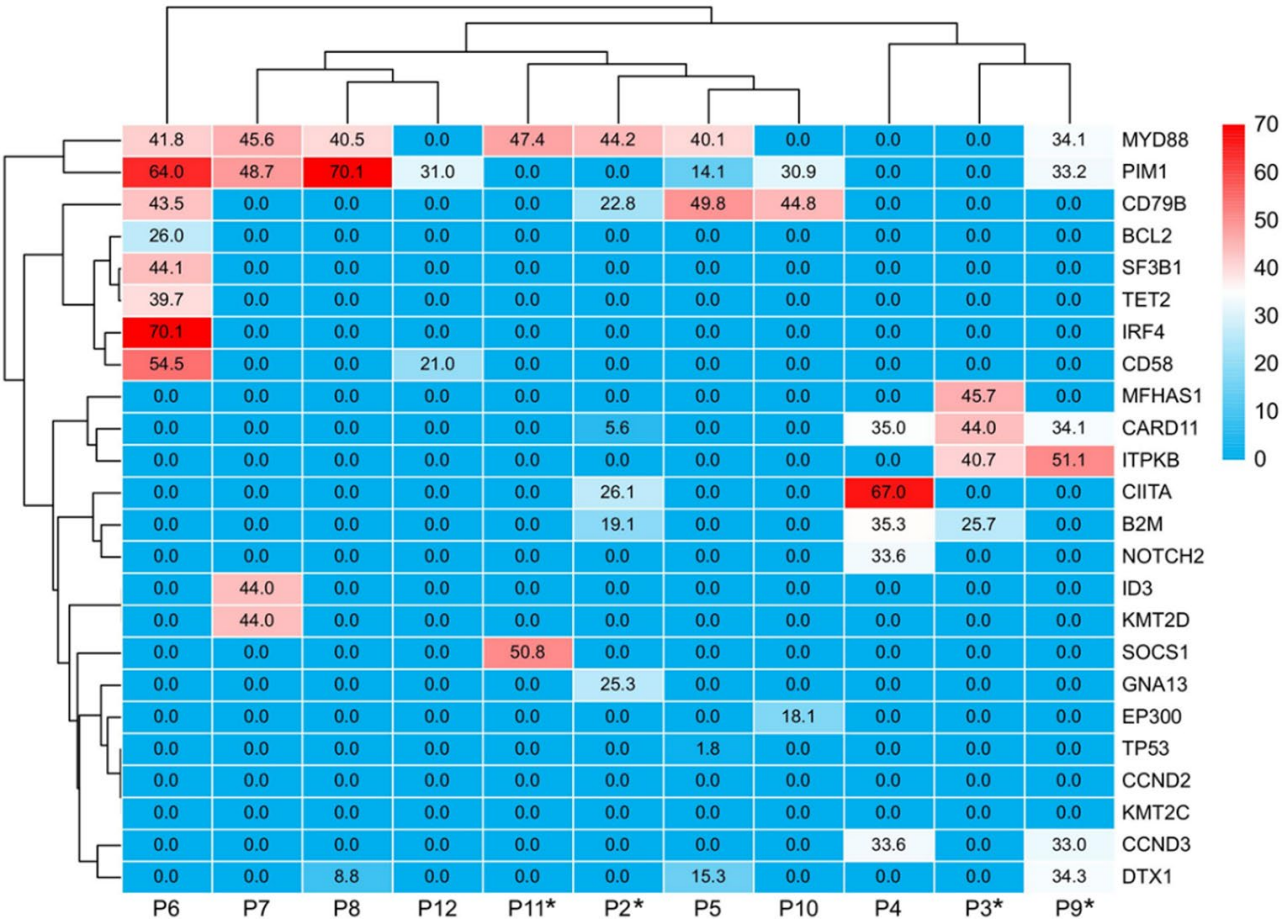
Genomic analysis using the next-generation sequencing. Each value represents the variant allele frequency (VAF) expressed as a percentage. “*” indicates patients with PD/Relapse

Baseline tumour biopsy samples from 11 patients were subjected to next-generation sequencing (NGS), which revealed common class I mutations in PIM1 (7/11), MYD88 (7/11), CD79B (4/11), and CARD11 (4/11). Among the 5 patients with PD/relapse, 4 patients had evaluable NGS data (“*” in Fig. 3). Notably, one patient with late relapse presented initial mutations in MYD88 and SOCS1, whereas three other patients presented heterogeneous nucleotide variants in CARD11 exon 6. Intriguingly, an additional patient with a CARD11 exon 8 mutation achieved PR during induction therapy and subsequently attained CR after 3 months of lenalidomide maintenance therapy. The heatmap clustering results are shown in Fig. 3.

### Safety

No patient discontinued treatment because of adverse events (AEs). The most frequently observed AE was neutropenia (41.7%, 5/12 patients), with all cases being grades 1–2 in severity. Other treatment-related AEs included cutaneous rash (25.0%, grades 1–2), transient aminotransferase elevation (16.7%, grade 1), and lower limb myalgia (8.3%, grade 1). The overall safety profile was favourable, with no grade 3 or higher toxicities reported.

## Discussion

This was a long-term follow-up study to evaluate the efficacy of lenalidomide as a first-line treatment for PCNSL without ASCT consolidation. Our results demonstrated that patients receiving R2-MTX achieved superior outcomes in terms of induction response rates, PFS, and OS. Specifically, the R2-MTX regimen yielded an ORR of 91.7% (95% CI: 61.5–99.8%) and a complete response (CR) rate of 75.0% following induction therapy. Similarly, the phase 2 trial by Qian et al. reported a relatively high ORR of 88.2% and a CR rate of 76.5% after induction therapy [12]. The IELSG32 trial applied methotrexate, cytarabine, thiotepa, and rituximab (MATRix) for first-line treatment, with an ORR of 87% [13]. In contrast, the combination of R-MTX with cytarabine or temozolomide yielded a lower ORR [13, 14].

ASCT consolidation has further improved the ORR of first-line treatments, with a 7-year overall survival (OS) rate reaching 71% for ASCT consolidation following complete response to MATRix induction therapy [5]. Therefore, ASCT may be considered an important consolidation strategy to increase remission depth in patients who respond to R2-MTX induction therapy. Unfortunately, in this study, none of the patients underwent ASCT. The main contraindications to ASCT included patients being unsuitable for transplantation, patient refusal, and the high cost of thiotepa as a preconditioning drug, leading to limited accessibility.

This study featured an extended follow-up period (median: 43 months), during which the R2-MTX regimen demonstrated significant survival benefits. The 2-year overall survival (OS) rate of 91.7% observed in our cohort is comparable to the 90.4% reported in the phase Ib/II study by Zhang et al. [15] and exceeds the 69% achieved with the methotrexate–cytarabine plus rituximab and thiotepa regimen (Arm C) in the IELSG32 trial [13]. Notably, our long-term outcomes revealed estimated 5-year OS and PFS rates of 70.7% (95% CI: 52.1–99.3%) and 57.1% (95% CI: 34.5–83.7%), respectively, which were comparable to those achieved with the more intensive Arm C regimen [13]. However, we recognize the limitations of this study and the possible resulting bias caused by the small sample size.

In this study, one patient who achieved a PR prior to maintenance therapy achieved a CR after three months of maintenance therapy, which suggests that lenalidomide maintenance therapy may facilitate deeper remission. Although previous studies have reported no significant prognostic improvement with lenalidomide maintenance therapy in R2-MTX-treated PCNSL patients [16], our center’s experience contrasts these findings. In our cohort, patients who underwent R-MTX plus lenalidomide or ibrutinib induction, followed by maintenance therapy, demonstrated superior outcomes compared with those without maintenance therapy (unpublished data). Notably, one patient experienced late relapse 32 months after lenalidomide discontinuation but subsequently achieved a CR following salvage therapy with ibrutinib plus R-MTX and ASCT. This case highlights two key observations, as follows: (1) despite prolonged event-free survival, late relapse remains a concern with R2-MTX regimens, and (2) ibrutinib may retain clinical activity in lenalidomide-refractory patients.

All three patients who experienced early relapse were found to have mutations in exon 6 of the CARD11 gene. Another patient with a mutation in exon 8 of the CARD11 gene required a significantly prolonged time to achieve CR. Mutations in the CARD11 gene are relatively common in patients with PCNSL, accounting for approximately 10–16% of mutations [17, 18], and lead to persistent activation of the NF-κB signalling pathway [3]. This activation promotes the survival, proliferation, and immune evasion of tumour cells and is associated with the aggressive phenotype of PCNSL and a poor prognosis of patients [18]. Theoretically, lenalidomide can inhibit the NF-κB signalling pathway [19, 20], thereby reducing the signal activation caused by CARD11 mutations and suppressing the survival and proliferation of tumour cells. However, our results show that patients with CARD11 mutations still have a high rate of early relapse, even with lenalidomide treatment. This finding requires further verification in prospective studies. Additionally, the impact of different nucleotide or amino acid mutation sites on the prognosis of patients with CARD11 mutations is another issue that needs further investigation in the future.

In the IELSG-32 study, 7% of patients died because of treatment-related toxicity [13]. Similarly, in the PRIMAIN study, R-MP regimens with or without lomustine resulted in an 8.4% incidence of treatment-related fatalities [21]. Therefore, drug-associated toxicity is a critical concern that must be meticulously evaluated when selecting therapeutic options, particularly for older patients. In this study, the combination of lenalidomide and R-MTX demonstrated excellent treatment tolerability, and no patients who received R2-MTX treatment experienced grade 3 or higher adverse events. The most common adverse effects were neutropenia (41.7%) and rash (25.0%), which resolved following symptomatic management. Primary prophylaxis with G-CSF is suggested to prevent neutropenia in patients with DLBCL treated upfront with intensive immunochemotherapy regimens [22].

This study has several Limitations. First, the sample size was small, and the estimated 5-year survival rate may not have accurately reflected true survival outcomes. Second, our patients did not undergo ASCT; thus, the generalizability of the results may be limited. Third, this was a retrospective study. In the future, prospective multicentre studies with larger sample sizes will be essential to further validate the superior efficacy and to explore the beneficial factors of lenalidomide in newly diagnosed PCNSL patients.

In conclusion, the high response rate and good tolerance observed in this study suggest that the R2-MTX regimen is a potential therapeutic option for newly diagnosed PCNSL patients. Our data provide preliminary evidence that the R2-MTX regimen might be feasible for patients unsuitable for ASCT. These findings warrant cautious interpretation until they are validated in larger, prospective studies.

## Data Availability

No datasets were generated or analysed during the current study.
